# C-reactive protein as a prognostic factor in patients with chordoma of lumbar spine and sacrum—a single center pilot study

**DOI:** 10.1186/s12957-016-0875-8

**Published:** 2016-04-18

**Authors:** Gerhard Martin Hobusch, Florian Bodner, Sonja Walzer, Rodrig Marculescu, Philipp T. Funovics, Irene Sulzbacher, Reinhard Windhager, Joannis Panotopoulos

**Affiliations:** Department of Orthopaedics, Medical University of Vienna, Waehringer Guertel 18 – 20, A-1090 Wien, Austria; Department of Medical and Chemical Laboratory Diagnostics, Medical University of Vienna, Vienna, Austria; Clinical institute of Pathology, Medical University of Vienna, Vienna, Austria

**Keywords:** CRP, Chordoma, Survival, Recurrence, Prognostic marker, Resection margins, Inflammation

## Abstract

**Study design:**

This is a retrospective, diagnostic study, level IV.

**Background:**

It appears to be necessary to identify prognostic markers for individual risk estimation for progression and survival in patients with chordoma, a rare disease. Are pre-operative serum levels of C-reactive protein (CRP) associated with disease progression and survival?

**Methods:**

Survival rates of 24 patients (18 males, 6 females) (mean age 67 years (SD ± 16; range 20–85 years); minimum follow-up 2 years, mean follow-up 5 years (SD ± 5; range 2–19 years)) with chordoma of the lower spine and sacrum were assessed with a focus on pre-operative CRP levels.

**Results:**

The survival rate of patients with pre-operative CRP level of >1.0 mg/dl was lower than that of patients with a CRP level <1.0 mg/dl (*p* = 0.01). The estimated 10-year survival of patients with pre-operative CRP values <1.0 and >1.0 mg/dl was 76 and 25 %, respectively. CRP remained as an independent survival factor (*p* = 0.025; CI 95 % 1.0–2.6) in multivariable analysis.

**Conclusions:**

Pre-operative CRP levels appear to be a biomarker for disease-specific survival in patients with chordoma of the lumbar spine and sacrum. A validation of our finding with larger cohorts and integration of putative risk factor would further elucidate CRP a surrogate for tumor progression.

## Background

Chordoma is one of the most common, primary malignant bone tumors of the spine. About 50 % of these tumors occur in the caudal spine, pelvis, and sacrum [1]. It is a rare neoplastic disease (0.9/1,000,000 citizens/year) [2] arising from embryonic notochord. Large databases reveal 5-year survival rates between 67 and 78 % [1, 3, 4]. Tumor metastases are scarce; nevertheless, its primary local aggressive growing leads to significant morbidity.

In previous clinical research, several prognostic factors for an inferior survival have been speculated. Age [5], tumor size >8 cm [6], the tumor site regarding the proximal extent of the tumor, and local invasion into other tissues [7] may influence survival [1, 8]. Local surgical resection of the tumor [1, 4, 6] improves survival if the localization involves the sacral bone but not the spine. Local recurrence is higher when inadequate margins are achieved [9–11]. Local recurrence is an important predictor of disease-related mortality [8, 12]. A more radical surgical approach results in a decrease of recurrence to a half or a third, at least in sacral chordomas [9, 13–15]; however, radical surgery compromises life quality mostly for these elderly patients. The abovementioned prognostic markers are mainly based on the clinical-pathological findings. Therefore, pre-operatively and reliable prognostic markers for individual risk estimation of recurrence and patient’s survival are needed.

In recent studies, the concept of the involvement of systemic inflammation in cancer progression has been postulated [16]. Specifically elevated C-reactive protein (CRP) as a marker of systemic inflammation [17] has been found to be associated with decreased survival. C-reactive protein (CRP) is a plasma protein mainly produced in the liver and triggered by interleukin IL-6 [18]. An elevated serum level of CRP (>1.0 mg/dl) is associated with both an increased risk in all cancer [19] and a prognostic factor for survival and recurrence in different types of both carcinoma, including breast, prostate, colon, and hepatocellular carcinoma, and also bone and soft-tissue sarcoma [20–23] including osteosarcoma, Ewing’s sarcoma and chondrosarcoma. However, even minor elevations of CRP (>0.3 mg/dl) implicate adverse outcome on a variety of medical cardiovascular and non-cardiovascular conditions [24]. An association of CRP and a higher risk of death were seen in several cancers such as lymphoma, multiple myeloma, esophageal carcinoma, and colorectal cancer.

The impact on survival of pre-operative serum CRP levels in patients with chordoma has not yet been elucidated. This retrospective single-center study is an attempt to evaluate pre-operative CRP in context with this disease.

## Methods

A review of our Institute’s bone and soft-tissue tumor register revealed 34 patients ((24 males, 10 females) with a mean age of 66 years (SD ± 16; range, 20–85 years) and a consecutive mean follow-up (FU) of 5 years (SD ± 6; range, 2–19 years)) with diagnosed chordoma of the lumbar spine and sacrum at our department from 1985 to 2012. The inclusion criteria for this retrospective study were histologically verified chordoma and a minimum follow-up of 2 years, unless death from the disease had occurred within these 2 years. Pre-operative in-house assessment of C-reactive protein was required to avoid sample bias. Therefore, 10 patients had to be excluded. Histological specimens were analyzed within the first 3 weeks postoperatively; all were primary chordomas. The clinical records of the remaining 24 patients (18 males, 6 females) (mean age 67 years (SD ± 16; range, 20–85 years); mean FU 5 years (SD ± 5; range 2–19 years)) were carefully reviewed. These patients form the base of this study.

Tumor site in 5 (20 %) patients was in the lumbar spine and in 19 (80 %) patients in the sacrum. The distribution of localization relative to S3 included 14 (58 %) chordoma distal of S3 and 10 (42 %) chordoma proximal of S3. Histologic diagnosis was confirmed by morphology and immunohistochemical positivity of S-100, CAM 5.2, and FU 5 at the local institute of pathology of every bioptic specimen prior to surgery. Tumors were subclassified into chondroid, conventional, and dedifferentiated chordoma. All patients had resections of the tumor including 1 (4 %) incomplete vertebrectomy, 3 (12.5 %) total vertebrectomies, 18 (70 %) partial sacrectomies, and 3 (12.5 %) resections of the coccygeal bone. Resection margins were wide in 10 (42 %) patients, marginal in 12 (50 %) patients, and intralesional in 2 (8 %) patients according to the Enneking criteria [25]. In seven patients, postoperative radiotherapy was administered when chordoma was localized in the lumbar spine or in case of an intralesional resection. Two patients received an irradiation of 50 Gy, four patients a dose of 60 Gy, and one patient a dose of 66 Gy each in two Gy EDs doses. Confounding diagnoses and conditions known to be as well associated with increased CRP were assessed, including all known tumors and cardiovascular risk factors (see Table 1). These were taken into account as confounding factors and were taken covariates in the multivariable testing model.

**Table 1:** Demographics of patients with chordoma

Number	Sex	Age (years)	Histology	Tumor site	Follow-up	Death of disease	CRP (mg/dl)	Confounding diagnosis	
								Tumor	CVD
1	Male	68	Conventional	Prox S3	2.7	No	0.81		Hypertensive heart insufficiency II, Hyperlipidemia
2	Male	85	Conventional	Distal S3	11.1	No	0.10		Hypertension
3	Male	52	Conventional	Distal S3	6.3	No	0.22		Hypertension
4	Male	79	Conventional	Distal S3	3.7	No	0.85		Hypertension, type II diabetes, obesity
5	Male	81	Conventional	Distal S3	2.8	No	1.18	Prostataca	
6	Female	81	Conventional	Distal S3	6.0	No	2.24		
7	Female	79	Conventional	Distal S3	1.4	Yes	0.87		
8	Male	71	Conventional	Prox S3	8.9	No	0.40		
9	Male	82	Conventional	Distal S3	1.6	Yes	0.00		
10	Male	58	Chondroid	Distal S3	4.2	No	0.40		Hypertension, hyperlipidemia
11	Male	84	Conventional	Distal S3	4.1	Yes	1.10		
12	Male	56	Chondroid	Prox S3	8.1	No	0.15		Hypertension, obesity
13	Male	73	Conventional	Prox S3	2.1	No	0.70		
14	Male	35	Conventional	Distal S3	1.6	Yes	0.90		
15	Female	66	Conventional	Prox S3	3.6	No	0.58		
16	Male	62	Conventional	Prox S3	4.3	No	3.30		
17	Male	62	Conventional	Prox S3	3.0	Yes	3.10		
18	Male	20	Conventional	Distal S3	0.5	Yes	0.81		
19	Female	77	Conventional	Prox S3	16.5	No	1.32	Basalioma	
20	Female	77	Chondroid	Prox S3	3.0	Yes	0.14		
21	Male	60	Conventional	Distal S3	18.8	No	1.95		Hypertension
22	Female	68	Dedifferentiated	Prox S3	4.4	Yes	0.20		
23	Male	52	Conventional	Distal S3	2.1	No	7.60		Hypertension, renal insufficiency, chon. Prostatitis
24	Male	77	Conventional	Distal S3	1.3	Yes	8		

Tumors (additional tumors)

*CVD* cardiovascular disease (meant to be confounders of increased CRP)

Prior to this investigation, approval by the review board of the Medical University of Vienna was obtained and informed consent was routinely obtained from the patients for further use in their files and registry data for scientific purposes.

### C-reactive protein

Patients’ blood was obtained before tumor resection by venous puncture in 24 patients (18 males/6 females) with a mean age of 67 years (SD 16 years; range, 20–85) and a mean follow-up of 5 years (SD 7 years; range, 2–19). CRP measurements were not expressly ascertained for study purposes but performed as part of a pre-operative clinical routine by a latex-enhanced immunoturbidimetric test (Olympus AU 2700 CRP Latex, Beckman Coulter Inc., Brea, California) according to the manufacturer’s instructions just prior to surgery (day −1 or date of surgery). The respective hardware specifications indicate a coefficient of variation of 1.65–3.79 % in a normal sensitivity setting, covering serum CRP levels ranging from 0.02 to 45 mg/dl. Serum CRP levels were categorized into below 1 mg/dl and >1 mg/dl.

### Statistics

Statistical analyses of the data focused on disease-specific survival in relation to CRP levels. CRP values (mg/dl) are shown as means with standard deviation (SD). CRP (<1.0 mg/dl and >1.0 mg/dl) values were compared using an unpaired *t* test and Pearson correlation analysis. Demographic (sex and age), surgical (surgical method, tumor site, and resection margins), and clinical-pathological variables (serum CRP-level, death due to disease, recurrent disease) were examined. Age and pre-operative serum CRP and fibrinogen and alkaline phosphatase levels, as well as tumor size were regarded as continuous variables; all other covariates were modelled as categorical variables. Investigated endpoints of the study were death due to disease and recurrent disease. Survival probabilities were calculated with the Kaplan-Meier product limit estimator. To compare survival functions of two or more groups, we applied the log-rank test. Uni- and multivariable analyses were calculated by a Cox regression model. The multivariable model was calculated with the covariates, age, gender, histologic subtype, and possibly confounding diagnosis. All statistical tests were two-sided. All analyses and graphical visualizations were performed using the Statistical Package for the Social Sciences, Version 20.0 (SPSS, Chicago, Illinois). The alpha level for all tests was set at *p* = 0.05, and *p* < 0.001 was considered highly significant. A power analysis was performed using GraphPad StatMate (GraphPad Software Inc., LaJolla, CA; version 2.00, 2004) and revealed that the overall survival estimation comparing patients with CRP values above 1.0 mg/dl to those with normal values had a power of 0.45 to detect a minimal survival difference of 58 % with *p* = 0.05 (two-tailed). The respective survival difference observed in this study was 59 %.

## Results

The mean follow-up period was 5 years (median 4 years; range, 0.5–19 years). The mean follow-up of 15 patients surviving the disease was 6.7 years (median 6 years; range, 2–19 years). Nine patients died due to disease 2.3 years (median 2.1 years; range, 0.5–4.4 years) after surgery. Sixteen patients (67 %) developed recurrent disease/disease progression. Two patients developed metastatic disease including one skin metastasis of the thigh and one lung metastasis.

### C-reactive protein and survival

The mean pre-operative serum CRP level of all 24 patients was 1.22 mg/dl (SD 1.6 mg/dl). The pre-operative CRP of 16 patients was <1.0 mg/dl (62 %) and the CRP of eight patients >1.0 mg/dl (38 %).

A pre-operative CRP level >1.0 mg/dl was significantly correlated with death due to disease (*p* = 0.006, *r* = 0.548). Patients who died of the disease had higher pre-operative CRP values than survivors (mean 2.3 mg/dl, SD ± 2.2 mg/dl versus mean 0.5 mg/dl, SD ± 0.4 mg/dl) (*p* = 0.046).

**Fig. 1:**
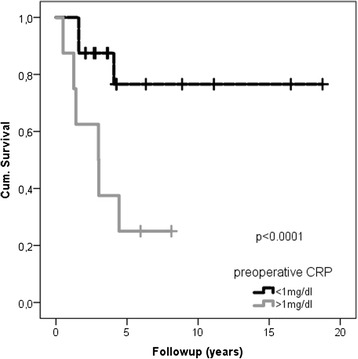
Kaplan-Meier curves overall survival categorized into pre-operative CRP levels <1.0 and >1.0 mg/dl

Patients with a pre-operative CRP level of >1.0 mg/dl had a lower survival rate than patients with a CRP level <1.0 mg/dl (*p* = 0.01) (Fig. 1). The estimated 2-, 5-, and 10-year survival of patients with pre-operative CRP values <1.0 mg/dl was 87, 76, and 76 % with >1.0 mg/dl 62, 25, and 25 % respectively.

CRP was the strongest prognostic factor of survival in univariable analysis (*p* < 0.0001) and remained significant regarding survival (*p* = 0.025; CI 95 % 1.0–2.6) in multivariable analysis.

### C-reactive protein and tumor progression

The mean CRP value of patients without recurrence was 0.6 mg/dl (SD ± 0.4 mg/dl; range, 0–0.9 mg/dl); the mean CRP value of patients who developed recurrence was 1.5 mg/dl (SD ± 1.1 mg/dl; range, 0.1–3.3 mg/dl). There was a trend in the difference between patients with CRP <1.9 and >1.0 mg/dl in context with recurrence (*p* = 0.09). The estimated 2-, 5-, and 10-year recurrence-free survival of patients with pre-operative CRP values <1.0 mg/dl was 87 %, 57 % and 46 % with >1.0 mg/dl 57, 14, and 0 % respectively.

In univariable analysis CRP was significantly associated with the incidence of tumor recurrence (*p* < 0.0001) (see Table 2) and remained significant regarding recurrence (*p* = 0.023; CI 95 % 1.0–2.3) in multivariable analysis.

**Table 2:** Univariable analyses of clinical and oncological factors with patient overall survival and recurrence

Variable	*p* value	*p* value
	Survival	Recurrence
Gender^a^	0.830	0.418
Age^b^	0.393	0.341
Tumor size^b^	0.64	0.668
Resection margins^a^	0.44	0.379
Site (lumbar or sacral)^a^	0.946	0.985
Site (proximal or distal S3)^a^	0.402	0.895
Histologic subtype^a, c^	0.821	0.974
RTX^a^	0.992	0.74
Possibly confounding diagnosis^a, d^	*0.003*	*0.024*
Recurrence^a^	*0.045*	*/*
Pre-operative CRP levels^b^	*<0.0001*	*<0.0001*
Pre-operative aP levels^b^	0.285	0.563
Pre-operative fibrinogen levels^b^	0.506	0.71

^a^Calculated using log-rank test

^b^Calculated using Cox regression

^c^Differentiation (conventional, chondroid, dedifferentiated)

^d^Tumors and CV risk factors were taken into account

## Discussion

Chordoma is a rare and challenging disease with many possible sequelae. Surgically, the radical resection was considered, to the best of our knowledge, as state-of-the-art treatment, but there are upcoming new therapeutical options, i.e., proton radiation. Surgical margins in chordoma resection still seem to be the utmost decisive factor concerning longer patient survival. However, the last and new century brought new perspectives regarding tumor biology, microenvironment, and tumor surveillance, and we are looking for prognostic markers to estimate survival before manipulating our patients. The concept of systemic inflammation (i.e., baseline CRP) has been described to be important for disease-prognostic purpose in many malignancies. In bone sarcomas, both the survival [26–30] and recurrence have shown significant associations with pre-operative CRP values [20–23]. To the best of our knowledge, pre-operative CRP values have not yet been associated with outcomes for chordoma patients. Therefore, our aim was to identify an association between pre-operative CRP values and the survival in this rare disease.

The limitations of this study are certainly its retrospective design and the low number in the respective groups compared, owing to the low incidence of this rare disease, thereby reducing the statistical power of Kaplan-Meier analyses to 0.45 and implying utmost critical interpretation of the conclusions deducted. Given the present incidences of death in the group of patients with CRP levels above 1.0 mg/dl, a power of 0.8 would require a sample size of 14 patients with CRP levels above 1.0 mg/dl, thereby increasing the required patient group by a factor of 2.3 to an estimated total of 49 patients, a number rarely reported even in large scale studies.

Furthermore, the small number of patients was due to our criteria to only include patients with a complete FU and with in-house assessment of the CRP. As the CRP is related to a lot of other clinical situations, as well as tumors, this study was not designed to find patients with tumors but to find patients with a high likeliness of a poor outcome, which may be related to higher amount of CRP. The covariate “confounding diagnosis” in the multivariate analysis was introduced to take into account the influence of other diseases on CRP as one factor.

In this presented study, CRP has the strongest prognostic value for overall survival of all tested factors. A CRP above 1.0 mg/dl was significantly linked to death due to disease, and death due to disease mostly occurred after two and a half years.

Several prognostic factors have been described to influence the outcome of chordoma patients. Recognized prognostic parameters such as age, large tumor size, and the localizations above S3 are all known to lead to a poor outcome. We could not prove that age and localization of tumor were co-factors for survival in our collective. However, a trend towards low survival and big tumor size was identified. Resection margins were significantly associated with recurrence, which in consequence was a significant prognostic factor for survival. A wide en bloc resection with the removal of the biopsy tract should be performed according to several authors [9, 13–15]; however, radical surgery is often not feasible. In consequence, prognostic factors could help physicians confronted by the reality of these difficult decisions. One large-sized attempt for a pre-operative disease-prognostic grading scale in chordoma patients revealed age and tumor-invasion as independent prediction factors, but blood parameters were not taken into account [7]. Known prognostic factors for adverse survival and recurrent disease are mostly assessed after surgery. Determination of serum CRP is possible prior to surgery, and moreover, it is cheap, feasible, and part of routine-laboratory assessment in almost every medical care unit.

There is a growing knowledge regarding inflammation and its complex interplay with cancer. As CRP is known as an acute-phase-protein in inflammation and many types of cancer develop in a chronic-inflammatory microenvironment, it may appear possible that infection directs the processes of tumor growth, recurrence, and metastatic disease [31]. CRP is mainly produced in hepatocytes and is regulated by different interleukins (IL-6, IL-1β). Il-6 is produced by macrophages [32] and is known to be associated with tumorigenesis, cancer growth, and invasion. Recently, CRP expression was enhanced in vascular smooth muscle cells after exposure of mechanical strain and IL-6 [33]. This may explain that local expansion in chordoma and any other kinds of tumor leads to higher CRP levels in more aggressive tumors by mechanical forces.

Even minor CRP elevations show a trend towards low survival in this cohort of chordoma patients. These minor CRP elevations may express a mild degree of tissue stress, reflecting the presence of distressed cells according to Kushner et al. [24].

## Conclusions

To conclude, this finding of pre-operative CRP levels as a prognostic marker in disease-specific survival for patients with chordoma may be an important aspect for individualized treatment in this disease that is characterized by high recurrence and morbidity. A multicentered approach involving CRP and other biomarkers to be tested in settings with combined treatment including surgery and other upcoming treatment options, i.e., carbon ions [34] based on individual risk of patients could be the next step to find new treatment algorithms for chordoma.

## Abbreviations

CRP: C-reactive protein
FU: follow-up
SD: standard deviation
